# Fifty years at the addiction bench and bedside: from withdrawal syndromes to adaptive brain states

**DOI:** 10.3389/fpubh.2026.1969403

**Published:** 2026-10-27

**Authors:** Mark S. Gold

**Affiliations:** 1 Department of Psychiatry, University of Florida, Gainesville, FL, United States; 2 Department of Psychiatry, Washington University in St. Louis School of Medicine, St. Louis, MO, United States; 3 Department of Psychiatry, Tulane University School of Medicine, New Orleans, LA, United States

**Keywords:** addiction, buprenorphine, cocaine, dopamine hypothesis, naltrexone, neuroplasticity, recovery, substance use disorder

## Abstract

Over the past half-century, addiction medicine has moved from moral and symptom-based explanations toward a neuroscience of adaptation, learning, motivation, and recovery. This Perspective uses several linked translational programs—opioid withdrawal and the locus coeruleus, cocaine dependence and dopamine, environmental drug exposure, physician health, and food reward—to illustrate how observations at the bedside can generate mechanistic studies and how laboratory findings can, in turn, change treatment and public health. LC stimulation produced signs and symptoms that looked to me like opioid withdrawal. So, we thought that noradrenergic hyperactivity in opioid withdrawal caused symptoms, and while methadone or morphine could treat these, we could also treat them with a non-opioid, clonidine. Earlier noradrenergic interventions had been attempted with limited clinical success. Our work provided the first hypothesis-driven, double-blind, placebo-controlled demonstration that a non-opioid medication could suppress the acute human opioid withdrawal syndrome. This mechanism-based treatment was greeted with considerable interest because some clinicians had assumed that any medication capable of reversing opioid withdrawal distress must itself be an opioid. Clonidine and later lofexidine did just that and facilitated transition to naltrexone. Recognition that cocaine could produce compulsive use without a dramatic physical withdrawal syndrome helped shift the field toward reward circuitry, craving, pathological attraction, anhedonia, and relapse. Studies of tobacco, cannabis, and opioid smoke broadened prevention by showing that involuntary second- and third-hand exposure can be biologically detected and, most clearly for tobacco, can produce biologically meaningful effects. Third-hand exposure refers to residual drug-related contaminants that persist on indoor surfaces, dust, clothing, or other materials after active smoking has ended. Contemporary work on neuromodulation, exercise, psychedelics, and GLP-1 metabolic-reward pathways extends the same translational logic. Addiction is best understood as an acquired adaptive brain state supported by anti-survival or bad learning, development, and environment; recovery is active neurobehavioral relearning, not simply detoxification or drug clearance. Public health should therefore join brain sciences, work to delay initiation, identify emerging risk before a severe substance use disorder develops, prevent involuntary exposure, and provide rapid access to mechanism-based treatment, recovery-supportive environments, and outcomes measured over 5 years or longer.

## 1 Introduction: from moral judgment to mechanism

Long before addiction became a medical specialty, physicians and writers recognized compulsive alcohol use. Greek and Roman observers described intoxication, habitual drunkenness, and the physical and moral consequences, while Shakespeare portrayed alcohol’s ability to overwhelm judgment and transform behavior. In early America, Benjamin Rush—a physician, signer of the Declaration of Independence, and later recognized as the father of American psychiatry—argued that habitual drunkenness was a disease requiring treatment rather than simply a moral defect. He also published the first American textbook on mental illness in 1812. Yet, despite these early insights, addiction remained outside mainstream medicine well into the twentieth century.

When I entered neuroscience and addiction research in the early 1970s, addiction was commonly viewed as the consequence of personality disorder, weak or defective character, or social deviance. Clinical definitions emphasized intoxication, tolerance, and visible withdrawal. The consequences were immediate: many patients with addiction were excluded from ordinary medical settings, prevention relied heavily on moral and religious admonition, and treatment remained largely separated from mainstream medicine.

When I decided to devote my career to addiction neuroscience and treatment development, my Psychiatry Chairman tried to persuade me that this was a childish career choice. He pointed out that my mentor—one of the few recognized addiction experts—did not even have an office in the medical center. In his view, addiction was not a legitimate medical specialty or a serious field of scientific inquiry. The field, he told me, did not exist. In many ways, he was right. My career would be devoted to helping build it.

The scientific change that came was not simply the replacement of a moral disgust model by a “brain disease” slogan. It was the accumulation of neuroscience evidence that repeated exposure changes interacting systems for reward, stress, learning, memory, salience, motivation, and executive control. Addiction is not due to one transmitter or one brain region, but neither can its behavioral features be understood without the brain. Modern reviews now describe addiction as a disorder involving durable circuit and behavioral transformations, formed by early access and development, environment, and access to effective care (1, 2).

This Perspective traces an addiction-science revolution that was slowed for decades by the scarcity of scientists, physicians, treatment programs, and research resources devoted to addiction. Progress was possible with translational work in which I participated and through closely related advances by others. It is not a systematic review or a claim that any one laboratory created the modern field. Rather, it argues for a recurring addiction science method: begin with a clinically important observation, identify a testable mechanism, move across experimental levels, return to patients, and then translate the result into treatment, prevention, education, and policy.

## 2 Opioid withdrawal: a new circuit linked to distress becomes a treatment target

Opioid withdrawal provided an early demonstration that a syndrome commonly interpreted as psychological distress could be mapped onto a specific neurobiological mechanism. My work in the early 1970s at the University of Florida with Al Rhoton, Steve Zornetzer, and Bill Luttge introduced me to the rodent and human nucleus locus coeruleus (LC) (3) and the Mayo Clinic studies of Pozuelo and Kerr (4). After deciding against a career in neurosurgery, I went to Yale to do neurosurgical and other interventional work with rodents and nonhuman primates (5) and translate findings in humans as part of the behavioral sciences training program. It was a smooth transition, as work in Aghajanian’s laboratory focused on the LC, the brain’s principal noradrenergic nucleus, in opioid dependence and withdrawal. Converging cellular, nonhuman-primate, and clinical observations supported the hypothesis that abrupt opioid cessation produces noradrenergic hyperactivity and that reducing this activity should suppress withdrawal (6–11).

Clonidine, an alpha-2 adrenergic agonist, tested that proposition. In patients, clonidine reduced major autonomic and psychological components of acute opioid withdrawal without acting as an opioid replacement (6, 12–14).

Once we could detoxify patients with OUDs, we worked on transitioning them to a long-term treatment program with Naltrexone at its core. Subsequent work developed rapid non-opioid detoxification strategies and paired clonidine with naltrexone, helping patients move from withdrawal management to antagonist-based relapse prevention (15–17). These studies did not make detoxification sufficient treatment; contemporary care properly emphasizes ongoing medications for opioid use disorder, retention, overdose prevention, and long-term recovery support (18, 19). Their greater significance was methodological: a defined neural mechanism could help explain opioid actions and withdrawal while yielding a clinically useful, non-addictive intervention. The same mechanism subsequently led to lofexidine, an alpha-2 adrenergic agonist that reduced opioid withdrawal with fewer sedative and hypotensive effects than clonidine in early clinical studies (20). Later controlled trials confirmed its efficacy (21), and in 2018 the FDA approved lofexidine as the first non-opioid medication specifically indicated for mitigation of opioid withdrawal symptoms in adults. This regulatory endpoint was a direct descendant of the circuit-based treatment hypothesis developed through the earlier clonidine studies.

This bench-to-bedside sequence also illustrates an important safeguard. Mechanistic elegance is not the same as clinical completeness. Clonidine treats withdrawal signs and symptoms; it does not by itself treat the full chronic disorder. Naltrexone outcomes were particularly favorable among physicians and other professionals whose return to safety-sensitive employment was contingent on treatment adherence, monitoring, and continued abstinence (22). Their experience demonstrated that medication effectiveness depends not only on pharmacology but also on retention, accountability, and a recovery environment that supports adherence. Public health systems fail when they treat detoxification as an endpoint instead of an entry to continuing care. The translational lesson is therefore threefold: mechanisms can explain clinically important features of addiction, mechanistic insight can improve acute care, and acute stabilization must be connected to durable treatment and long-term recovery support (19, 23, 24).

## 3 Addiction Studies in Physicians and Executives

Physicians and business executives offered an important natural experiment. Anesthesiologists were overrepresented among physicians treated for opioid use disorders and were at particular risk for overdose despite education, income, housing, employment, access to health care, and detailed knowledge of drug risks (25, 26). Business people and celebrities had access to cocaine and resources to buy it. These wealthy patient cohorts did not eliminate every social or psychological influence, but they reduced socioeconomic disadvantage as the dominant explanation for addiction. They also allowed us to ask whether mechanism-based treatment could work in people whose recovery could be followed objectively and whose professional functioning mattered to patients, families, and institutions.

We included addicted physicians and executives in the early clonidine studies and in the transition from clonidine-assisted opioid withdrawal to naltrexone (27). This work helped establish a practical sequence: control noradrenergic withdrawal without another opioid, confirm that the patient was opioid-free, and begin an opioid antagonist to reduce the risk that renewed opioid use would be reinforcing. In the pre-fentanyl era, these studies helped make naltrexone clinically usable and contributed to the evidence base that preceded its 1984 approval, as Trexan, for opioid dependence. They similarly reinforced a lesson that remains current: detoxification is valuable when it opens the door to sustained relapse-prevention treatment, not when it is treated as an endpoint.

State medical boards generally permitted physicians to return to practice only when they remained in good standing with a Physician Health Program (PHP) and followed the contracted treatment plan. The physician cohort had a 5-year contract, and with the Board of Medicine’s help they became a model for long-term recovery management. PHPs combined evaluation and treatment with peer role modeling, professional accountability, mutual-help participation, work-site oversight when needed, and monitoring contracts commonly lasting 5 years. Participants called the PHP (which by itself was a treatment) each workday to learn whether they had been randomly selected for an observed urine test; failure to call was itself clinically meaningful. In the national study of 904 physicians, a distinctive cohort of licensed professionals under 5-year contracts whose livelihoods were contingent on compliance, 78% had no positive alcohol or drug test during prolonged monitoring, and 72% were still licensed and practicing without evidence of substance misuse or malpractice 5 to 7 years after entering their contracts (28, 29). Recurrence prompted rapid reevaluation, intensified treatment, and closer monitoring rather than automatic abandonment. My mentee Lisa Merlo continued this line of research, examining physician impairment, the essential elements of successful monitoring, and the durability of recovery (28, 30).

Physicians, including anesthesiologists, know more than most people about pharmacology, overdose, and dependence, yet knowledge alone did not protect them from addiction. We knew this because they were over-represented in treatment in Florida and elsewhere. Information is therefore necessary but is not prevention by itself. Effective prevention must also address access, exposure, stress, reinforcement, early identification, protective environments, and rapid routes to confidential treatment. The physician model showed that addiction is not confined to poverty or lack of education. At the same time, its outcomes showed that sustained structure and monitoring can convert recovery from an aspiration into a measurable long-term process. The experiences of physicians with addiction and the national data on their treatment also changed how I thought about prevention.

## 4 Cocaine: addiction without dramatic physical withdrawal

Cocaine forced a second conceptual revision. In the late 1970s and early 1980s, it was considered a nonaddictive “champagne of drugs” because its discontinuation did not resemble alcohol or opioid withdrawal. DSM-III, published in 1980, did not yet recognize cocaine dependence as a distinct addictive disorder in the form later adopted by DSM-III-R. Patients nevertheless reported binge use, anhedonia, craving, compulsive drug seeking, and repeated unsuccessful efforts to stop. These observations demonstrated that physical withdrawal could not define addiction.

Before Charles Dackis and I developed the cocaine dopamine-depletion hypothesis, I was already collecting the clinical evidence that cocaine was addicting. Beginning in 1983, the national 800-COCAINE information and referral service allowed us to interview thousands of people who called because cocaine had taken control of their lives. Over time, the service assisted more than 2 million callers. The data documented a recognizable progression from use to compulsive use; craving and inability to stop; and substantial psychiatric, medical, family, occupational, and financial consequences. They also identified a changing epidemic, including increasing proportions of women and progressively younger and lower-income callers. Most importantly, the callers confirmed that cocaine abuse was an addictive illness even without the dramatic autonomic withdrawal syndrome associated with opioids or alcohol (31). The results helped overturn the prevailing myth that cocaine was nonaddictive and contributed to the clinical evidence underlying formal recognition of cocaine dependence in DSM-III-R in 1987.

The next question was mechanistic: if cocaine produced addiction without classic physical withdrawal, what had changed in the brain? Work with Charles Dackis and colleagues proposed the dopamine-depletion hypothesis: repeated or binge cocaine exposure was followed by altered dopaminergic function that contributed to dysphoria, reduced reward, impaired motivation, and renewed use (32–35). The specific pharmacologic implications of that early hypothesis evolved—dopamine agonists such as bromocriptine did not become established treatments—but its central move proved durable. Volkow and colleagues subsequently tested the Dackis-Gold hypothesis with PET, demonstrating reduced dopamine D2-receptor availability associated with reduced frontal metabolism that persisted after detoxification, while refining rather than simply confirming the original receptor prediction (36). Cocaine dependence became understood as a disorder of reward and motivation rather than a series of intoxications. This reframing converged with independent theories emphasizing reinforcement rather than physical dependence, opponent and anti-reward processes, and sensitized incentive salience (37–39). Later molecular, behavioral, and human imaging work elaborated this into models of reduced reward sensitivity, conditioned salience, impaired inhibitory control, and persistent vulnerability to relapse (1, 2, 40). The 800-COCAINE experience also established a public-health principle that remains current: information is most useful when it joins surveillance and education to an immediate route into treatment. Representative examples of this recurring translational sequence are summarized in Table 1.

**Table 1:** Five decades of translational addiction research: from clinical observation to enduring principle.

Clinical observation or problem	Translational program and contribution	Clinical or public-health consequence	Enduring insight
Opioid withdrawal resembled experimental LC activation	LC noradrenergic hyperactivity; clonidine and later lofexidine	First non-opioid treatment shown under double-blind, placebo-controlled conditions to suppress acute opioid withdrawal in humans	A withdrawal syndrome can be mapped to a circuit and treated without replacing the drug
Detoxification alone did not prevent recurrence	Sequential clonidine-to-naltrexone treatment	Connected acute withdrawal relief with antagonist-based relapse prevention	Detoxification is a bridge to continuing treatment—not treatment completion
Physicians developed addiction despite knowledge, status, and resources	Physician Health Programs; five-year monitoring, peer support, accountability, and rapid reintervention	High rates of sustained recovery and return to practice	Knowledge alone does not prevent addiction; recovery improves with duration, structure, purpose, and measurement
Cocaine produced compulsive use without dramatic physical withdrawal	800-COCAINE surveillance and referral; dopamine-depletion hypothesis	Helped establish cocaine dependence as an addictive disorder and connected callers with care	Addiction is defined by pathological reinforcement, craving, and impaired control—not physical withdrawal alone
Some drug-related changes persisted after the drug had cleared	Methamphetamine neurotoxicity; overdose-related hypoxic injury, imaging, and neuropsychological studies	Expanded outcomes beyond abstinence to cognition, function, and rehabilitation	Reversible neuroadaptation must be distinguished from persistent brain injury
Nonsmokers and children showed detectable involuntary drug exposure	Tobacco, cannabis, opium-smoke, and occupational fentanyl-exposure research	Supported environmental measurement, biomonitoring, ventilation, and exposure prevention	Secondhand exposure is firsthand exposure for the person who did not consent
Patients substituted food, sugar, or other rewards for drugs	Food-addiction models; Avena-Hoebel studies; Brownell conference; Gearhardt and the YFAS	Connected addiction neuroscience with compulsive eating and obesity research	Addiction can involve narrowing of reward without classic withdrawal or an illicit drug
Metabolic treatments appeared capable of modifying drug reward	Bidirectional addiction-obesity research and GLP-1 receptor agonists	Generated potential new approaches for alcohol and other substance use disorders	The gut, metabolism, and brain reward systems form an integrated treatment target
Recovery involved more than drug removal	Adaptive-brain-state model; medication, psychotherapy, exercise, sleep, mutual help, meaningful work, and monitoring	Encourages multidimensional, long-term outcome measurement	Recovery restores reward diversity: one drug → many rewards

## 5 From transmitter theories to the adaptive-brain-state model

Dopamine matters, endogenous opioids matter, endocannabinoids matter, and stress systems matter. Yet no single system explains why drug cues become powerful, natural rewards lose value, behavior narrows, and relapse risk can persist after drug clearance. The more complete account is neuroadaptation: repeated exposure recruits learning and memory, reward and antireward processes, stress reactivity, habit formation, and executive control.

I describe addiction as an acquired adaptive brain state. “Adaptive” does not mean healthy or chosen. It means that the nervous system changes its operating conditions in response to repeated exposure. Tolerance, withdrawal, craving, and cue reactivity are different expressions of that altered state. Developmental timing matters because exposure during adolescence and emerging adulthood occurs while prefrontal, limbic, and social-learning systems are still maturing (41). Drug potency, route, frequency, co-exposures, trauma, social context, and commercial availability determine what is learned and how strongly.

This scheme also clarifies recovery. Abstinence removes the drug but does not instantly restore sleep, reward valuation, mental adaptability, social trust, or a workable daily life. Recovery requires new learning: cues lose predictive strength; non-drug rewards regain salience; routines become less rigid; relationships and purpose provide competing reinforcement. The physician health experience described above makes the more general principle visible: recovery is supported by time, structure, meaningful roles, objective measurement, and fast response to recurrence.

This view avoids two opposite errors. Addiction is not just a bad choice, but neither are people passive victims of disordered circuitry. Neuroplasticity is the mechanism through which addiction is learned and also one mechanism through which recovery becomes more stable. Recovery is rebuilt slowly, day after day, in which choices add up to support healthy biological, behavioral, and social living. The relationship between addiction and recovery as competing adaptive brain states is summarized conceptually in Figure 1.

**Figure 1:**
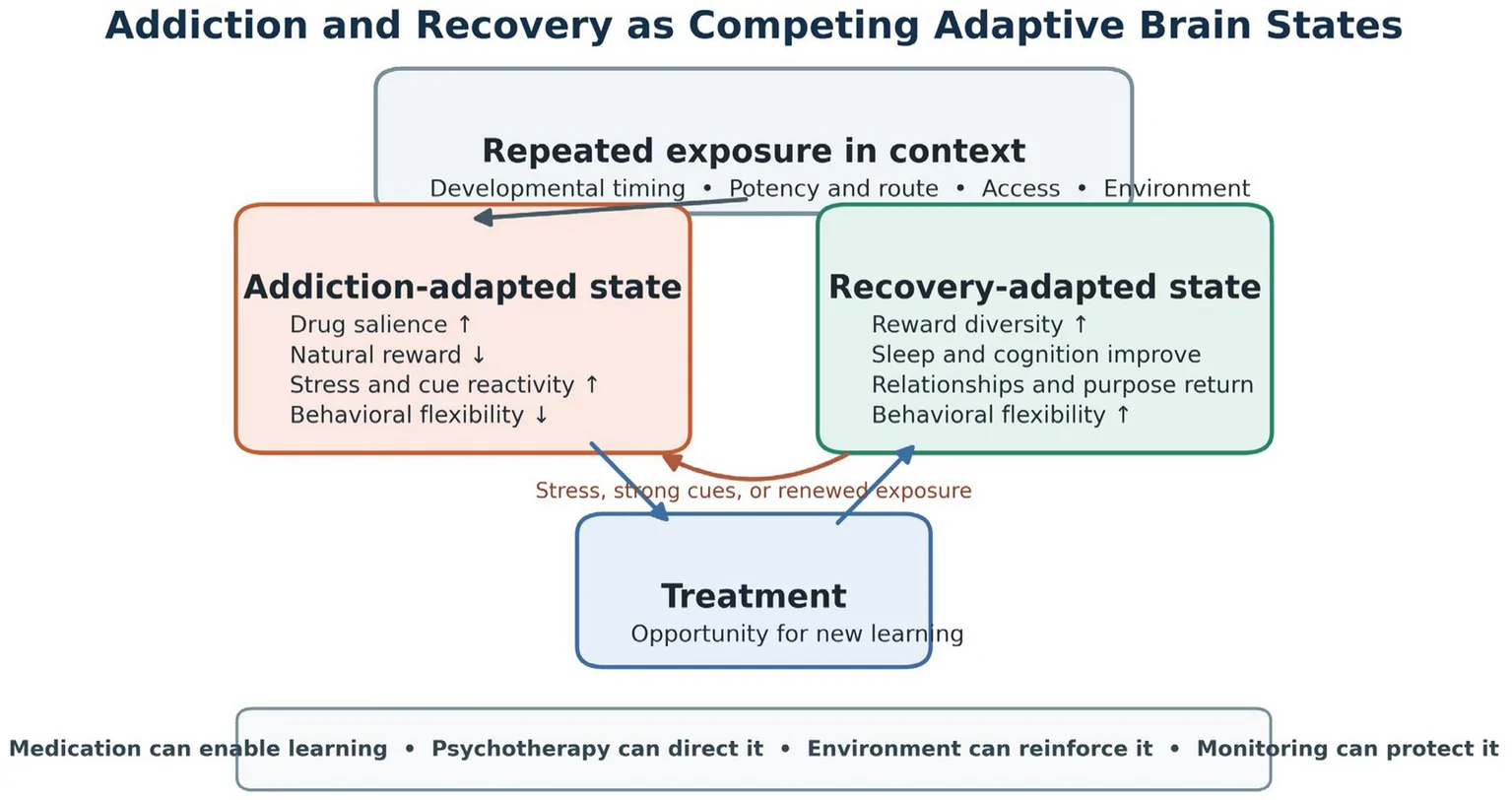
Addiction and recovery as competing adaptive brain states. Repeated exposure, developmental timing, drug characteristics, access, and environment can produce an addiction-adapted state marked by increased drug salience and cue reactivity, reduced natural reward, and diminished behavioral flexibility. Detoxification removes the drug but does not automatically reverse this learned state. Treatment and recovery-supportive environments create opportunities for relearning, restoration of reward diversity, and stabilization of a healthier adaptive state. Stress, cues, or renewed exposure can reactivate vulnerability, making monitoring and rapid response to recurrence important. This is a conceptual model; neither state is unitary or irreversible.

## 6 Reversibility, persistent injury, and return of function

The adaptive-brain-state model carries a hopeful implication: many functional changes can improve with abstinence, treatment, restored sleep, exercise, cognitive engagement, and sustained recovery. Yet reversibility should not be assumed to be complete or uniform. In work with Jean Lud Cadet and colleagues at NIDA, we compared methamphetamine-related injury with traumatic brain injury and emphasized overlapping oxidative, inflammatory, excitotoxic, apoptotic, and necrotic pathways (42). Longitudinal rodent imaging likewise demonstrated persistent methamphetamine-associated changes in brain structure and function (43). Some neurological and neuropsychological effects may recover over months or years, whereas others—particularly after heavy cumulative exposure, psychosis, trauma, vascular events, or polysubstance use—may persist. Treatment studies should therefore measure cognition and real-world function, not only drug use or craving. Opioid intoxication adds a distinct and often overlooked mechanism of injury. Repeated “nodding,” prolonged respiratory depression, and nonfatal overdose can produce hypoxic-ischemic or anoxic injury; survivors may develop memory, attention, executive, motor, or delayed white-matter syndromes even after naloxone restores breathing (44). These events should be documented and tracked as clinically important comorbidities, with neurological examination, neuroimaging or serial neuropsychological assessment when indicated, and referral for cognitive, physical, occupational, or other neurorehabilitation. The long-term goal is not merely abstinence but the greatest possible return toward premorbid function. Psychedelic-assisted treatments and agents such as ibogaine may eventually contribute to that goal.

Still, current ibogaine evidence includes promising uncontrolled observations rather than proof of neural restoration. Cherian and colleagues studied 30 male Special Operations Forces veterans, predominantly with mild traumatic brain injury rather than a substance use disorder, in a prospective open-label protocol that used magnesium to mitigate QT-prolongation risk and reported no clinically meaningful arrhythmia or hemodynamic instability (45). Cardiac risk nevertheless remains a major concern in the wider ibogaine literature. Future trials should distinguish reversible adaptation from structural injury and test whether treatment improves durable cognition, function, and quality of life.

## 7 Prevention as protection from involuntary and developmental exposure

Environmental exposure research enlarges the unit of analysis from the individual user to families, workplaces, and communities. Translational animal models showed that tobacco smoke can supply nicotine sufficient to produce dependence-related effects (46), while controlled nicotine-withdrawal studies demonstrated measurable deficits in brain reward function (47). Cannabis-smoke models likewise demonstrated biologically active cannabinoid exposure and behavioral change (48). These experiments established a general principle: route and dose determine pharmacology; the exposed brain does not distinguish whether exposure was intentional.

Human studies extended that principle beyond the laboratory. A technical report from Afghanistan detected opium products in air and on household surfaces in homes where opium was smoked, documenting second- and third-hand exposure among women and children (49). In operating rooms, our secondhand-exposure hypothesis proposed that repeated low-level exposure to aerosolized fentanyl or other anesthetic agents might contribute to sensitization or occupational vulnerability among anesthesiologists (50, 51). This is still a hypothesis rather than a complete explanation of anesthesiologist addiction, which is multifactorial, but it generated testable questions about ventilation, environmental measurement, biomonitoring, access, and workplace protection.

The same logic applies to developmental prevention. Prevention should not rely exclusively on remote harms. For adolescents, a more accurate message is that repeated exposure may train a developing brain to assign disproportionate value to one rapid and reliable source of reward.

Prevention protects the capacity to learn broadly—from relationships, education, exercise, mastery, and work—before behavior and reward become narrowed.

## 8 Exceeding traditional drug categories

The transition from drug addiction to behavioral addiction did not begin as a theoretical leap. It began with cocaine. For years, patients described binge use, craving, anhedonia, loss of control, and repeated relapse. At the same time, many authorities continued to doubt that cocaine was truly addicting because it lacked the prominent physical withdrawal seen with opioids or alcohol. Putting together the clinical observations, dopamine studies, animal models, and treatment data took years. Once that evidence was accepted, addiction could no longer be defined by dramatic withdrawal alone. Its more general features were compulsive reinforcement, narrowing of reward, continuation despite harm, and impaired control.

That change in definition created the bridge to gambling and other behaviors. If a substance could produce addiction without a classic withdrawal syndrome, then repeated behaviors capable of powerfully engaging reward, learning, anticipation, and cue-driven motivation could also be studied using addiction criteria. Gambling became the clearest accepted example. The implication was not that every pleasurable or repetitive behavior is an addiction, but that the absence of an ingested drug—or of prominent physical withdrawal—could no longer end the scientific research. The relevant questions evolved into loss of control, functional impairment, continuance despite consequences, craving or preoccupation, and repeated unsuccessful efforts to stop.

Food and sugar followed this same translational path. The clues first came from patients: cigarette smokers gained weight after quitting; cocaine users often forgot to eat; patients withdrawing from opioids craved sweets; and people in residential recovery programs frequently developed overeating and rapid weight gain. These observations suggested that drugs and highly palatable foods could substitute for one another and recruit overlapping systems of reward and reinforcement (52–57).

Bart Hoebel’s laboratory at Princeton provided the experimental bridge. Work with Nicole Avena showed that intermittent access to sugar could produce bingeing and dependence-like behavioral and neurochemical changes in animal models. As Avena’s mentor on her NIDA K01 Career Development Award, I helped frame a translational program aimed at developing new treatments for obesity by applying medications and principles already used in addiction treatment to hedonic overeating and food addiction (54, 55). At that stage, we expected the therapeutic direction to run primarily from addiction medicine toward obesity. Kelly Brownell then helped bring leading addiction and obesity scientists together at the 2007 Yale Historic Conference on Food and Addiction. Those discussions contributed to our Oxford handbook, Food and Addiction, while Ashley Gearhardt gave the field a standardized human measure through the Yale Food Addiction Scale (58, 59). This progression—from bedside observations, to animal models, to measurable human phenotypes—helped establish food and highly processed foods as legitimate subjects of addiction research rather than metaphors.

The food-addiction debate remains especially important and appropriately contested. Food is necessary for life, abstinence is impossible, and eating is influenced by metabolism, culture, availability, stress, and social determinants. The strongest addiction analogy applies not to all food or all obesity, but to patterns of compulsive consumption involving highly rewarding, rapidly absorbed, highly processed products engineered and marketed for repeated use. The adaptive-state framework therefore asks when reinforcement becomes a persistent, clinically impairing narrowing of behavior—not whether pleasure itself is pathological. Similar operational caution is needed in studying problematic internet use and compulsive sexual behavior (60, 61).

This history also explains why metabolic treatments now matter to addiction science. Our early strategy was to ask whether addiction treatments—including naltrexone, bupropion, topiramate, and related approaches—could reduce hedonic eating and obesity. In retrospect, we should also have asked the question in the opposite direction: could medications developed for weight loss and type 2 diabetes treat alcohol and other substance use disorders? GLP-1 receptor agonists now make that possibility biologically plausible. Developed for diabetes and obesity, they act on an integrated gut-brain system influencing appetite, reward, craving, reinforcement, and decision-making. Early clinical studies imply possible effects on alcohol consumption and other addictive behaviors (62). The signals remain preliminary and require rigorous trials. Still, the conceptual movement is now clearly bidirectional: addiction neuroscience helped explain compulsive eating and generate obesity treatments, while obesity and diabetes biology are now generating possible treatments for substance use disorders.

## 9 The next translational frontier

Several emerging approaches fit the same bench-to-bedside logic. Neuromodulation attempts to alter circuits involved in craving and control; a recent review of reviews found promising but heterogeneous evidence across invasive and non-invasive modalities (63). Exercise can influence glutamatergic signaling, stress regulation, neuroplasticity, and alternative reinforcement, while preclinical work suggests that timing and intensity may change cocaine-related learning (64, 65). Psychedelic-assisted treatments may open time-limited periods of altered salience, emotional processing, or plasticity, but enthusiasm must not outrun trial quality, expectancy controls, safety monitoring, and evidence of durable benefit in substance use disorders (66, 67).

These approaches should not be divided into “biological” versus “behavioral” treatment. Medication can make new learning possible; psychotherapy can direct it; exercise, sleep, relationships, mutual-help groups, and meaningful work can reinforce it; policy can determine whether any of these are accessible. The next generation of trials should therefore measure more than short-term abstinence. Outcomes should include reward diversity, sleep, cognition, function, quality of life, overdose, recurrence severity, and treatment retention.

## 10 Momentum and hope for the future

The contrast with 1975 is extraordinary. When I began this work, the practical medication armamentarium was essentially limited to disulfiram for alcohol dependence and methadone for opioid dependence. Access to either was uneven, treatment was commonly isolated from academic medicine, and most medical schools offered little addiction education. There were few academic addiction experts, no mature nationwide system of fellowship-trained addiction physicians, and little expectation that treatment could be selected according to mechanism, comorbidity, stage of illness, or individual biology. Many patients and families were told, implicitly or explicitly, that little could be done.

Today, patients can receive evidence-based medications for alcohol, opioid, and tobacco use disorders; overdose prevention; withdrawal management; behavioral treatments; recovery support; and longitudinal monitoring. Addiction medicine and addiction psychiatry are recognized subspecialties, supported by accredited fellowships, board certification, and professional communities including the American Society of Addiction Medicine (ASAM) and the American Academy of Addiction Psychiatry (AAAP). Specialists are increasingly available in academic medical centers, hospitals, emergency departments, primary care, and recovery programs. Access still is far from adequate, but addiction is no longer scientifically or professionally homeless.

The next step is personalized addiction treatment. Rather than asking only which drug a person uses, clinicians will more frequently integrate route and intensity of exposure, withdrawal and craving phenotype, sleep, pain, trauma, cognition, psychiatric and medical comorbidity, metabolic status, genetics, social environment, and stage of recovery. Treatment may then combine medication, psychotherapy, contingency management, mutual-help participation, exercise, sleep restoration, digital monitoring, or neuromodulation according to the mechanisms maintaining that individual’s adaptive state. Personalization should not mean expensive technology for a few; it should mean choosing the right evidence-based intervention, at the right intensity and time, for each patient.

Several emerging approaches make that future tangible. GLP-1 receptor agonists link metabolic signaling with reward and have produced encouraging, though still preliminary, human signals in alcohol use disorder (62). Orexin-receptor antagonism may influence arousal, sleep, stress, craving, and withdrawal; early human work with suvorexant supports continued study in opioid use disorder and polysubstance use (68). Psychedelic-assisted treatments may create time-limited opportunities for psychological and neuroplastic change, while invasive and non-invasive neuromodulation may help recalibrate circuits involved in craving, salience, and control (63, 66, 67). Other possibilities include longer-acting formulations, vaccines and monoclonal antibodies against selected drugs, medications targeting stress and glutamate systems, and combinations designed around co-occurring pain, depression, obesity, or sleep disturbance.

None of these investigational approaches is a shortcut, and none should displace treatments already known to reduce mortality and improve recovery. Hope must remain disciplined by randomized trials, safety monitoring, durability, access, and attention to who is excluded from research. Yet the direction is unmistakable. In 1975, the main challenge was convincing medicine that addiction was treatable. The challenge now is to make an expanding science of treatment available to every patient and to match that science more precisely to the person. After 50 years, I have more reason for optimism than at any earlier point in my career.

## 11 Discussion and public health implications

Five conclusions follow from this history.

First, bedside observations remain a source of discovery. Patients recognized cocaine addiction before prevailing definitions did. A health system that listens carefully can detect new syndromes earlier.

Second, mechanism matters when it changes action. The LC model was valuable because it generated a treatment; dopamine models were valuable as well. After all, they expanded the definition of addiction past physical dependence.

Third, detoxification is not treatment completion. Acute withdrawal care should be designed as a bridge to medications when indicated, psychosocial treatment, recovery support, and continuing measurement.

Fourth, exposure is a public health variable. Developmental stage, commercial potency, route of administration, and involuntary smoke or aerosol exposure deserve the same attention as individual diagnosis.

Fifth, recovery is an active adaptive process. The goal is not simply one drug removed but a life in which many rewards, relationships, and roles can compete successfully with the drug.

This account has limitations. It is a selective narrative Perspective centered on programs in which the author participated, not a systematic history of addiction science. The cited literature is intentionally weighted toward those translational programs and early mechanistic models were necessarily incomplete several emerging treatments discussed here remain investigational.

One benefit of 50 years in this field is pattern recognition: learning to recognize both a prospective therapeutic signal and the familiar ways in which enthusiasm can outrun evidence. That perspective led the American Psychiatric Association to ask me to organize a Council report evaluating the risks and potential psychiatric benefits of cannabis and cannabinoids. Published in the American Journal of Psychiatry, it concluded that evidence did not support prescribing cannabinoids for psychiatric disorders and emphasized risks in susceptible populations (69). Kevin Hill and I subsequently joined colleagues in extending that evaluation spanning the controlled-trial evidence for the therapeutic use of cannabis and cannabinoids in a JAMA review (70). The same pattern prompted colleagues and me to organize an early review of ketamine misuse, diversion, cognitive effects, and urinary and other medical consequences in 2016—nearly a decade before nonprescribed ketamine use became a more visible public health concern (71).

Caution, however, should not become therapeutic conservatism. I have additionally embraced treatments when converging clinical and circuit evidence justified moving forward. Findings from deep brain stimulation helped make it plausible that less invasive neuromodulation, particularly transcranial magnetic stimulation, might reduce anhedonia and craving and help patients with addictions and co-occurring psychiatric disorders. The evidence remains heterogeneous, but TMS and related approaches extend a mechanistic treatment tradition rather than departing from it (63). I view psychedelics similarly: recent reviews suggest genuine potential for selected substance use disorders, while also showing the need for better controls, careful patient selection, integration, safety monitoring, and durable outcomes (66, 67). Pattern recognition does not mean predicting that every innovation will succeed. It means knowing when to warn, when to investigate, and when the evidence is strong enough to move carefully toward patients.

Across five decades, the substances and technologies changed, but the central translational question did not: how does repeated exposure change the brain and behavior, and how can that knowledge improve prevention, treatment, and recovery? Can we reverse the changes and damage done and return the person to their premorbid level of function? The most useful answer is neither moral condemnation nor biological reductionism. Addiction is an acquired adaptive brain state expressed in a person, a developmental period, and an environment. Recovery is the construction of a healthier adaptive state. Public health succeeds when it makes that construction possible.

## Data Availability

The original contributions presented in the study are included in the article/supplementary material, further inquiries can be directed to the corresponding author.
